# Postoperative Thrombotic Thrombocytopenic Purpura after Total Hip Arthroplasty: A Case Report and Review of the Literature

**DOI:** 10.1155/2018/9716170

**Published:** 2018-10-23

**Authors:** Nick N. Patel, Jason A. Shah, Christopher K. Sadlack

**Affiliations:** Department of Orthopaedic Surgery, Emory University School of Medicine, Atlanta, GA 30329, USA

## Abstract

Thrombotic thrombocytopenic purpura (TTP) is an uncommon and potentially lethal microangiopathy that carries a >90% mortality rate if not treated urgently and appropriately. Postoperative TTP after orthopaedic procedures is particularly rare with only four case reports existing in the literature. We present the case of postoperative TTP in a 57-year-old female who underwent elective total hip arthroplasty. We believe this case adds to the limited literature surrounding the topic. While rare, TTP after orthopaedic procedures poses a real and potentially fatal condition if not managed appropriately. Therefore, it is essential for orthopaedic surgeons to be cognizant of postoperative TTP for timely diagnosis and treatment.

## 1. Introduction

Thrombotic thrombocytopenic purpura (TTP), initially described by Moschcowitz in 1925, is a rare but life-threatening microangiopathy characterized by uncontrolled thrombotic phenomena [1, 2]. The pathophysiology is most commonly thought to be dysfunction of ADAMTS13, a protease of von Willebrand factor (vWF) which plays a critical role in hemostasis through platelet aggregation [2]. Therefore, increased circulating vWF multimer levels found in TTP is associated with platelet-rich small vessel thrombi and hemolytic anemia. Concomitant thrombotic organ damage can sometimes be seen with renal, cardiac, and nervous system manifestations. Untreated TTP carries a significant mortality rate of >90%; however, timely appropriate treatment has reduced this rate to 10–20% [3]. The two main etiologies of TTP are inherited and acquired with the latter being more common and triggered by conditions such as infection, pregnancy, cancer, HIV, and lupus [4].

Occurrence of TTP after surgery is rare but has been described in the literature following vascular, cardiac, and gastrointestinal procedures [5–8]. TTP after orthopedic surgery is even more exceedingly rare with only four case reports existing in the literature to our knowledge [9–12]. We present the case of a patient who developed postoperative TTP after routine total hip arthroplasty and discuss pertinent issues regarding the identification and management of the condition.

## 2. Case Report

**Figure 1:**
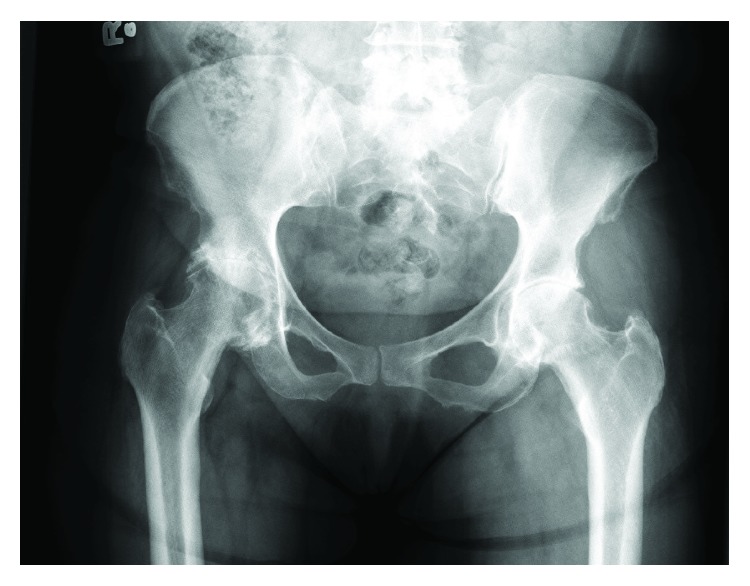
Preoperative AP pelvis radiograph demonstrating advanced degenerative changes of the right hip.

**Figure 2:**
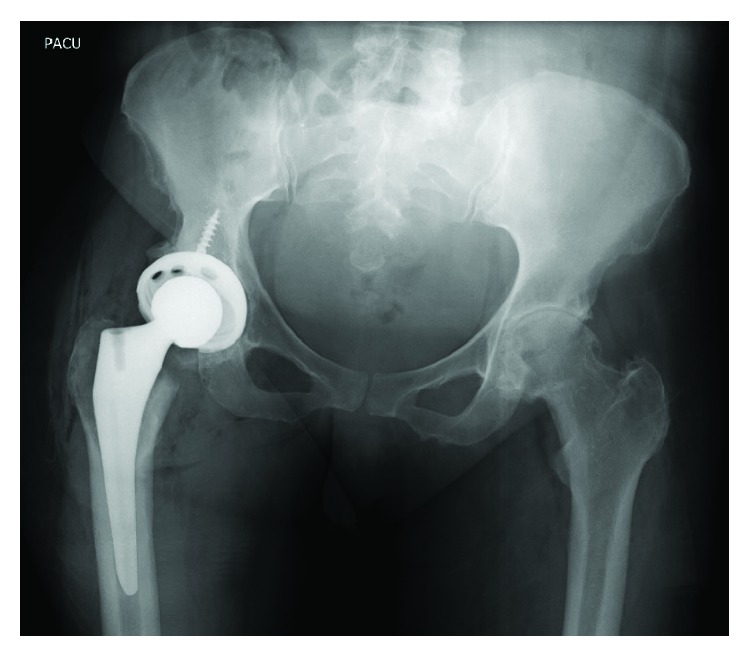
Postoperative AP pelvis radiograph after the right total hip arthroplasty.

At initial presentation, the patient was a 57-year-old female with body mass index (BMI) of 25.6 kg/m^2^ and medical history including controlled hypertension and asthma. Past surgical history consisted of three unremarkable caesarean sections. Her chief complaint was that of progressive right hip pain and radiographs demonstrated advanced osteoarthritis (Figure 1). After failing conservative measures, the patient underwent elective right total hip arthroplasty (Figure 2). Her surgery was uncomplicated with 200 cc of estimated blood loss. The patient had a preoperative hemoglobin of 13.5 g/dL and 13.3 g/dL on postoperative day one. Postoperative platelet count was also within normal limits at 231,000/mcL. She had an unremarkable hospital course and was discharged on postoperative day one.

The patient presented to the emergency room seven days after discharge with significant fatigue and shortness of breath with minimal exertion over the past day. She reported feeling nauseous with several vomiting episodes. Her husband also described one episode of confusion and word finding difficulty the previous day. What particularly alarmed the patient was the development of a yellowish appearance of her skin and eyes. The patient denied any complaints of her right hip.

Physical examination revealed a well-healing right hip surgical incision without evidence of hematoma, erythema, or drainage. She was noted to have scleral icterus, jaundiced palms, and scattered petechiae and purpura throughout her bilateral arms. Labwork demonstrated severe thrombocytopenia with a platelet count of 6,000/mcL and microcytic anemia with a hemoglobin of 5.4 g/dL and mean corpuscular volume (MCV) of 76 fL. Schistocytes were observed on peripheral blood smear. The hematology service was consulted given these abnormalities and high suspicion for TTP. The patient was transferred to the medical intensive care unit, and emergent plasmapheresis and steroids were initiated. Three units of packed red blood cells were transfused with appropriate rise in hemoglobin. The patient was found to have ADAMST13 activity of <3% (ref. range 68–163%) and positive ADAMST13 inhibitor titer confirming the diagnosis of TTP. A total of six plasmapheresis treatments were performed over the subsequent seven days. Following plasmapheresis treatment, the patient's platelet count steadily rose to a level of 468,000/mcL on the sixth day after readmission. Platelet transfusion was held as she demonstrated no clinical evidence of active bleeding during hospitalization. On subsequent days after initiation of treatment, the patient reported feeling much improved. Throughout her presentation, the patient maintained adequate urine output with creatinine level within normal limits suggesting no renal dysfunction. Laboratory values significantly improved (Table 1), and she was discharged on day six after readmission. At subsequent clinic follow-ups, the patient has been found to be doing very well with good ambulatory status, healed surgical wounds, and hematologic values within normal limits.

## 3. Discussion

Postoperative TTP is a rare but potentially lethal phenomenon if not correctly recognized and managed. Attention to patient history, physical exam, and labwork are crucial in order for accurate and timely diagnosis. As was seen in this case, TTP secondary to surgery routinely occurs 5–9 days postoperatively [13]. The patient in this case manifested three of the classically described pentad of symptoms associated with TTP which include thrombocytopenia, hemolytic anemia, fever, renal dysfunction, and mental status alteration. Caution must be taken to not falsely attribute these findings to other causes such as blood loss, hemodilution, or infection.

As was previously discussed, the pathogenesis for TTP is thought to be a dysfunction in the ADAMST13 enzyme [2]. In postoperative TTP, there is felt to be an additional contribution from endothelial damage at the time of surgery. This leads to extensive release of vWF multimers which overwhelm ADAMST13 available for cleavage [13]. Assays to quantify ADAMST13 activity and detect anti-ADAMST13 antibodies exist and were utilized in the care of this patient. These laboratory tests, however, can take multiple days to result and are not available at all institutions. Therefore, treatment should be promptly initialized based on clinical suspicion in order to decrease patient mortality.

Plasma exchange therapy in conjunction with adjuvant steroid administration has become the mainstay treatment for TTP and has shown to significantly reduce mortality [14]. Despite the presence of thrombocytopenia, platelet transfusion is generally withheld in the absence of clinically relevant bleeding [15]. Our patient's severe thrombocytopenia (6,000/mcL) steadily improved with the initiation of plasmapheresis to a platelet count of 468,000/mcL six days after treatment. Platelet count and lactate dehydrogenase level (LDH) have been shown to be useful markers for monitoring disease activity and, therefore, should be observed closely [16]. While our patient did not demonstrate evidence of renal dysfunction or other end organ damage, it is important to be aware of these potential findings.

Postoperative TTP following orthopaedic surgery is exceedingly uncommon as only four prior documented cases exist [9–12]. This case adds to the limited literature surrounding the topic and presents some unique features. While rare, TTP after orthopaedic procedures poses a real and potentially fatal condition if not treated appropriately. Therefore, it is essential for orthopaedic surgeons to be cognizant of postoperative TTP for timely diagnosis and treatment.

## Figures and Tables

**Table 1:** Laboratory values with reference ranges on the day of readmission and six days subsequently on discharge.

	Ref. range	Day of readmission	Date of discharge (6 days after readmission)
Hemoglobin	11.5–15.5 g/dL	5.4	9.8
MCV	80–97 fL	76	85
Platelet count	140,000–440,000/mcL	6,000	468,000
Serum LDH	91–180 U/L	1,189	256

MCV: mean corpuscular volume; LDH: lactate dehydrogenase; U: units; fL: femtoliter.
